# A dynamic interface between ubiquitylation and cAMP signaling

**DOI:** 10.3389/fphar.2015.00177

**Published:** 2015-09-04

**Authors:** Laura Rinaldi, Maria Sepe, Rossella Delle Donne, Antonio Feliciello

**Affiliations:** Dipartimento di Medicina Molecolare e Biotecnologie Mediche, University of Naples Federico II, Naples, Italy

**Keywords:** cyclic AMP, PKA signaling, proteasome, ubiquitination, AKAP

## Abstract

Phosphorylation waves drive the propagation of signals generated in response to hormones and growth factors in target cells. cAMP is an ancient second messenger implicated in key biological functions. In mammals, most of the effects elicited by cAMP are mediated by protein kinase A (PKA). Activation of the kinase by cAMP results in the phosphorylation of a variety of cellular substrates, leading to differentiation, proliferation, survival, metabolism. The identification of scaffold proteins, namely A-Kinase Anchor proteins (AKAPs), that localize PKA in specific cellular districts, provided critical cues for our understanding of the role played by cAMP in cell biology. Multivalent complexes are assembled by AKAPs and include signaling enzymes, mRNAs, adapter molecules, receptors and ion channels. A novel development derived from the molecular analysis of these complexes nucleated by AKAPs is represented by the presence of components of the ubiquitin-proteasome system (UPS). More to it, the AKAP complex can be regulated by the UPS, eliciting relevant effects on downstream cAMP signals. This represents a novel, yet previously unpredicted interface between compartmentalized signaling and the UPS. We anticipate that impairment of these regulatory mechanisms could promote cell dysfunction and disease. Here, we will focus on the reciprocal regulation between cAMP signaling and UPS, and its relevance to human degenerative and proliferative disorders.

## cAMP Signaling

**FIGURE 1:**
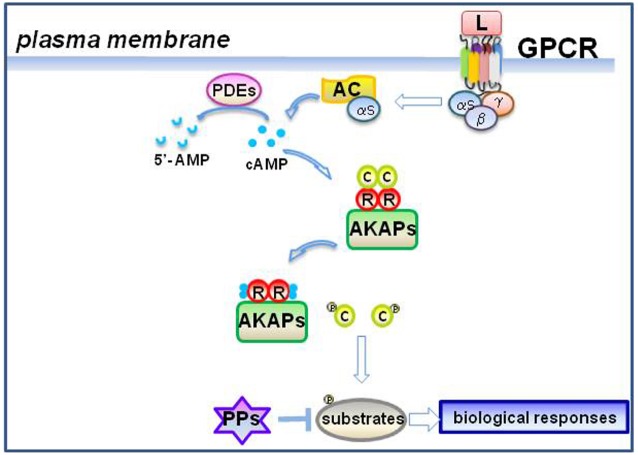
**GPCR stimulation and cAMP signaling.** Ligand-induced activation of a GPCR dissociates heterotrimeric G proteins and activates the adenylyl cyclase (AC) through Gαs subunit (Rosenbaum et al., 2009). AC converts ATP into cAMP. cAMP binding to regulatory (R) subunits of AKAP-assembled PKA dissociates the holoenzyme and activates the catalytic (C) subunits (Taylor et al., 2005). Phosphorylation of cellular substrates by C evokes plenty of biological responses. Phosphodiesterases (PDEs) converts the cAMP in 5′-AMP and decrease cAMP signaling (Maurice et al., 2014). Dephosphorylation of substrates by protein phosphatases (PPs) contributes to attenuate the signal (Zhang et al., 2013).

Since the discovery of cyclic adenosine 3′,5′-monophosphate (cAMP) in the late 1950s, significant advances have been made to better understand the link between the cAMP and the regulation of downstream signaling and cellular homeostasis. The principal elements of the cAMP cascade have been intensively studied, both at functional and structural side, delineating a complex and finely regulated network of signaling scaffolds and regulatory proteins (Walsh and Van Patten, 1994). cAMP levels are tightly regulated through the balance between two classes of enzymes: the adenylyl cyclases (ACs) and the cyclic nucleotide phosphodiesterases (PDEs). The main effector of cAMP is protein kinase A (PKA), whose role is fundamental in the propagation of the signal downstream to target substrates/effectors (Taylor et al., 2005). The duration and the amplitude of the propagating signal are controlled by a combination of different classes of ACs, protein kinases, PDE, phosphatases (PPs) and scaffold proteins (Figure 1).

G protein–coupled receptors (GPCRs) constitute a large family of membrane proteins that transduce signals from the extracellular microenvironment to inside cell (Rosenbaum et al., 2009). The binding of extracellular ligand to its cognate GPCR at the cell membrane activates AC, which in turn generates cAMP at discrete points along the plasma membrane. The mammalian ACs are encoded by nine independent genes differentially expressed in several cell types and tissues (Iyengar, 1993). The cAMP-generating activity of ACs is stimulated by the interaction with the stimulatory α subunit of the G-proteins (Gαs). In the absence of ligand, Gαs forms a heterotrimeric complex with the β and the γ subunits. Once activated, the GPCR causes the dissociation of heterotrimeric G-proteins, with consequent activation of ACs by the Gαs subunit (Cooper and Tabbasum, 2014). Continuous or repeated pulses of hormone stimulation downregulate GPCR activation. This phenomenon, called receptor desensitization (Reiter and Lefkowitz, 2006), includes two phases: (1) acute desensitization, which involves the recruitment of β-arrestin to the activated GPCR, impairing the coupling between the receptor and G-proteins; (2) long-term desensitization which consists in the internalization and lysosomal degradation of the receptors (Sibley and Lefkowitz, 1985; Bouvier et al., 1989; Moore et al., 2007). Although stimulation by Gαs is the major mechanism of AC activation, different isoforms of ACs can receive signals from a variety of sources, as kinases (PKA, PKC and Calmodulin kinase) or Ca^2+^, supporting and integrating distinct signal transduction pathways (Timofeyev et al., 2013; Zhang et al., 2013).

The cAMP-PDEs are enzymes that hydrolize the 3′,5′ phosphodiester bond in the second messenger cAMP, producing 5′-AMP (Maurice et al., 2014). By reducing the levels of cAMP, PDEs regulate the duration and amplitude of the cyclic nucleotide signaling. PDEs are encoded by 21 genes that generate 11 different families (PDE 1–11) that share structural similarities, but different substrate specificity, regulatory mechanisms and kinetics (Maurice et al., 2014). PDEs can hydrolyze cAMP (PDE4, PDE7, and PDE8), cGMP (PDE5, PDE6, and PDE9) or both cyclic nucleotides (PDE1, PDE2, PDE3, PDE10, and PDE11; Francis et al., 2011). The N-terminal regulatory region of PDEs controls the subcellular localization of the enzymes (Kenan et al., 2000). The differential distribution of PDEs within the cell generates intracellular microdomains of the second messenger that locally enhance the sensitivity and specificity of the signals carried out by cAMP (Lomas and Zaccolo, 2014). In this context, the use of cAMP biosensors, such as those utilizing fluorescence resonance energy transfer (FRET), contributed to dissect and visualize compartmentalized pools of cAMP that are generated in response to GPCR stimulation (Stefan et al., 2007). The establishment of the so-called “signalosome” is based on the protein–protein interaction network among the unique combinations of cyclic nucleotides generators (AC), effectors (PKA, EPAC, and cAMP-gated ion channels), degrading PDEs and scaffolds proteins (A-Kinase Anchor Proteins, AKAPs).

## Compartmentalized cAMP-PKA Signaling

In eukaryotes, most of the effects elicited by cAMP depend on the activation of PKA. This kinase consists of a tetramer composed of two regulatory (R) and two catalytic (C) subunits. The binding of cAMP to R subunit dissociates the PKA holoenzyme and releases the active C subunit, which in turn phosphorylates a wide array of cellular substrates, controlling different aspects of cell physiology (Taylor et al., 2008). The biochemical and functional features of PKA holoenzymes are largely determined by the structure, properties and relative abundance of the R subunits. The analysis of the kinetics of PKA activation/de-activation cycles contributed to understand the mechanisms of cAMP action on the effector kinase (Knighton et al., 1991). PKA stimulation by cAMP is followed by a refractory phase where a coordinated activation of Ser/Thr PPs, PKA inhibitors (PKIs) and changes in the ratio between R and C subunits eventually attenuate the signal (Armstrong et al., 1995; Canettieri et al., 2003).

The localization of PKA in the cell is mediated by scaffolding proteins, namely A-kinase anchoring proteins (AKAPs). AKAPs belong to a group of structurally different proteins that share the common feature to target the PKA holoenzyme in close proximity of its substrate (Michel and Scott, 2002). Each AKAP contains a PKA-binding motif that binds the R subunit of PKA and a targeting domain that directs the kinase to specific subcellular compartments. Biochemical and structural studies identified a conserved PKA-binding domain of AKAPs that forms an amphipathic helical wheel composed of 14–18-residues (Carr et al., 1992). The helical wheel binds with high affinity the N-terminal docking/dimerization (D/D) domain of the PKA-R dimer (Newlon et al., 1999, 2001). In particular, the hydrophobic residues of the helical wheel are located in the interior face, while charged residues align on the exterior surface. Although most of the AKAPs bind to RII subunit (Herberg et al., 2000; Carnegie and Scott, 2003), several RI-specific AKAPs have been characterized (Huang et al., 1997; Angelo and Rubin, 1998; Means et al., 2011; Burgers et al., 2012). The residues determining binding specificity of AKAPs to RI and RII have been partially defined (Alto et al., 2003). Disruption of the amphipathic helical wheel abrogates the binding to R subunits, both *in vitro* and *in vivo* (Welch et al., 2010). By modulating the dissemination of cAMP signals inside the cell, AKAPs control important biological responses, such as hormone secretion, metabolism, differentiation, cell growth and survival, synaptic transmission, learning and memory (Rubin, 1994; Alto et al., 2002; Tasken and Aandahl, 2004). AKAPs form a macromolecular complex, named transduceosome, that assembles components of cAMP generating systems (receptors and ACs), effectors (PKA and Epac) and attenuating enzymes (PDEs and PPs). This implies that complexes nucleated by AKAPs create intracellular domains where distinct signaling pathways converge and are locally attenuated or amplified, optimizing the biological response to extracellular stimuli (Feliciello et al., 2001, 2005; Dell’Acqua et al., 2006; Welch et al., 2010).

## Feed-backward Regulation of cAMP-PKA by the UPS

The ubiquitin-proteasome system (UPS) is emerging as an important control mechanism of cell growth, survival and metabolism. Degradation of a protein via UPS involves modification of the substrate protein by the covalent attachment of multiple ubiquitin molecules. The ubiquitin-tagged protein is eventually degraded through the proteasome (Ciechanover, 2005). Defects of the UPS may represent the trigger of several important human disorders (Wang and Hill, 2015; Dantuma and Bott, 2014; Ortega and Lucas, 2014; Schmidt and Finley, 2014). The ubiquitylation is mediated by the attachment of ubiquitin to the *ε*-amine of lysine residues of target proteins. This process requires a series of ATP-dependent enzymatic steps catalyzed by E1 (ubiquitin activating), E2 (ubiquitin conjugating), and E3 (ubiquitin ligating) enzymes (Ciechanover, 2003). The result of this sequential cascade of events is the covalent attachment of ubiquitin molecules to lysine residues on the target protein. These modifications can involve either a single ubiquitin (mono-ubiquitylation) or a chain of ubiquitin (poly-ubiquitylation; Ramanathan and Ye, 2012). Poly-ubiquitylation of a substrate is mostly related to protein degradation through the proteasome (Ciechanover, 2005). By modulating the protein levels, the UPS influences many cellular processes. Polyubiquitylated proteins can also follow a non-degradative pathway (De Bie and Ciechanover, 2011). This mechanism may control the intracellular trafficking of the target protein or its activity (Bonifacino and Weissman, 1998). In this case, de-ubiquitinating enzymes (DUBs), by removing the ubiquitin moieties, can restore the localization/activity of the modified protein (De Bie and Ciechanover, 2011).

**FIGURE 2:**
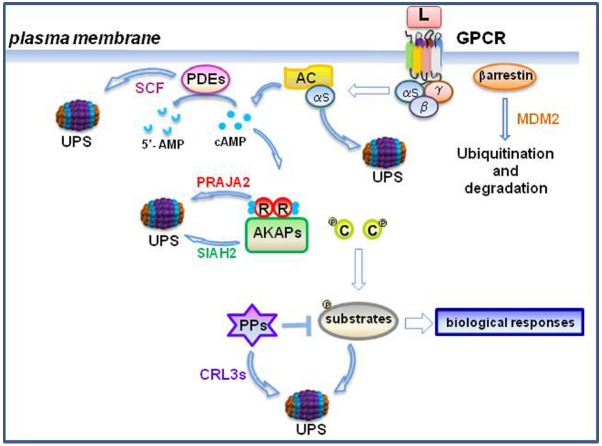
**Feed-back and feed-forward control of cAMP by the ubiquitin-proteasome system (UPS).** In the burst phase, ubiquitylation of cAMP-phosphodiesterases (PDEs) through the E3 ligase SCF complex contributes to modulate cAMP levels (Zhu et al., 2010). R subunits undergo to proteolysis by the praja2-UPS pathway. Loss of R subunits sustains PKA signaling (Lignitto et al., 2011). Moreover, ubiquitylation of protein phosphatases (PPs) by cullin E3 ligases (CRL3s) further modulates phosphorylation-dependent downstream signaling (Xu et al., 2014). During the desensitization phase, agonist-induced ubiquitylation of both receptor and b-arrestins promotes receptor endocytosis and degradation, attenuating downstream signaling (Reiter and Lefkowitz, 2006). Gαs subunits are ubiquitylated and degraded by the UPS (Zha et al., 2015). During hypoxia, Siah2-mediated ubiquitylation and proteolysis of AKAP121 modulates mitochondrial activity (Carlucci et al., 2008a).

The cAMP-PKA signaling is regulated by- and can regulate the UPS at different steps, giving rise to a complex interactive and regulatory network that controls different aspects of cell fate (Figure 2).

At cell membrane, the ubiquitylation and consequent proteolysis of receptors by the UPS contributes to post-stimulus receptor desensitization (Bonifacino and Weissman, 1998). As example, following β adrenergic receptor 2 (β-2AR) stimulation, the adaptor protein ARRDC3 (arrestin domain containing 3) recruits the E3 ligase NEDD4 (neural precursor development downregulated protein 4) close to β-2AR. Concomitant inhibition of the deubiquitinase USP20 (Ubiquitin-specific-processing protease 20) by PKA favors ubiquitylation and degradation of the receptor by NEDD4 (Nabhan et al., 2010; Kommaddi et al., 2015). Agonist-induced ubiquitylation of both receptor and β-arrestins (β-receptor regulatory proteins) also contributes to regulate receptor endocytosis. Internalized GPCRs can undergo to degradation or be recycled back to the cell surface (Shenoy et al., 2001). β-adrenergic signal transduction is the major pathway involved in the maintenance of cardiac muscle contraction. Reduced response to β-adrenergic stimulation and pathological cardiac hypertrophy are hallmark of heart failure (Port and Bristow, 2001; Tilley, 2011) In this context, blunted response to agonist might be a consequence of decreased levels of Gαs subunit (Tang et al., 2008). Accordingly, recent evidence indicates that in hypertrophic hearts Gαs undergoes to extensive ubiquitylation with suppression of its downstream signaling. This eventually leads to cardiac contractility dysfunction (Jenie et al., 2013). Gβ subunits can also become a target of the UPS, contributing to feed-back regulation of GPCR signaling. Thus, Gβ2 binds to DDB1 (DNA damage-binding protein 1), a core component of CUL4B-based E3 ubiquitin ligase complex, and targets the GPCR kinase 2 (GRK2) to ubiquitylation by the DDB1-CUL4A-ROC1 ubiquitin ligase complex. Following GPCR activation, PKA phosphorylates DDB1 and induces its dissociation from Gβ2, increasing the levels of GRK2 and promoting receptor desensitization. Deletion of Cul4a gene resulted in cardiac hypertrophy and this phenotype can be partially rescued by concomitant deletion of GRK2 (Zha et al., 2015). These results unvealed a novel mechanism of feedback regulation of GPCR signaling based on a non-canonical function of Gβ2 protein, that acts as a component of the ubiquitin ligase complex that targets GRK2 for degradation.

It emerged that PDEs can be regulated by the UPS. Ubiquitin conjugation and proteasomal degradation of PDE4D by a cullin 1-containing E(3) ubiquitin ligase complex is induced through concomitant phosphorylation of PDE4D by casein kinase 1 (CK1) and glycogen synthase kinase 3β (GSK3β). A phospho-degron motif within the PDE4D was identified as responsible of ubiquitin-mediated proteolysis of the enzyme. Interestingly, protein PPs calcineurin (CaN) counteracts the effects of the SCF complex on PDE4D stability (Zhu et al., 2010), unveiling a complex regulatory mechanism of signal integration between PPs and kinases involved in the control of cAMP pathway.

The UPS can also regulate the PKA stability and signaling. praja2 is a widely expressed mammalian RING-H2 protein with intrinsic E3 ligase activity (Yu et al., 2002). praja2 acts as an AKAP that binds and targets PKA holoenzyme to the cell membrane, perinuclear region and cellular organelles. Co-localization of praja2-PKA complexes with PKA substrate/effector molecules ensures efficient integration and propagation of the locally generated cAMP to distinct target sites. In course of agonist stimulation, praja2 couples ubiquitylation to proteolysis of the R subunits of PKA. By decreasing the ratio between R/C levels, praja2 sustains downstream signals carried out by PKA, positively impacting on specialized cell functions (Lignitto et al., 2011).

As major regulators of cAMP signaling, AKAPs can be regulated at post-translational level by the UPS. Thus, under normoxic conditions, mitochondrial AKAP121 assembles a multienzyme scaffold complex on the outer mitochondrial membrane that ensures efficient propagation of cAMP and src signals from sites of signal generation to mitochondria, enhancing oxidative phosphorylation, mitochondria remodeling, calcium homeostasis and cell survival (Cardone et al., 2004; Livigni et al., 2006; Dickey and Strack, 2011; Scorziello et al., 2013). Under hypoxic conditions, the RING E3 ligase seven in-absentia homolog 2 (Siah2) binds to- and ubiquitylates AKAP121. Ubiquitylated AKAP121 undergoes to proteasomal degradation. Disappearance of AKAP121 is accompanied by a significant drop of mitochondrial metabolic activity, leading to mitochondrial fission and cell death (Carlucci et al., 2008a,b; Merrill et al., 2011). In the ischemic mouse heart, infarct size and degree of cell death were blunted by genetic knock-out of Siah2. In hatching *Caenorhabditis elegans*, inhibiting Siah2 reduces life span, highlighting a role of the UPS-AKAP-PKA axis in the control of essential aspects of nematode aging (Kim et al., 2011).

## Feed-forward Regulation of the UPS by cAMP

Besides auto-regulatory mechanisms, cAMP can also control the activity of E3 ligases. By modulating the ubiquitin pathway, PKA controls the biological activity of a wide number of cellular substrates, integrating signals generated by distinct hormones/growth factors. As example, p300 acts as scaffold and co-activator for transcription factors, facilitating chromatin remodeling and gene expression. p300 controls important biological functions, as cell proliferation, differentiation, apoptosis, and senescence (Rack et al., 2014). In lung cancer cells, the levels of p300 are tightly regulated post-translationally by the cAMP signaling. Thus, agonist-induced rise of cAMP levels promotes ubiquitin-dependent proteolysis of p300, downregulating nuclear gene transcription (Jeong et al., 2013). The Ca^2+^/Calmodulin-dependent protein kinase III (CAMKIII), inhibits the elongation phase of translation by phosphorylating eukaryotic elongation factor-2 (eEF-2; Heise et al., 2014). Interestingly, CAMKIII protein levels are negatively regulated by isoproterenol stimulation of cAMP cascade. Degradation of the kinase requires the proteasome activity, linking the UPS to cAMP-dependent facilitation of protein translation (Wiseman et al., 2013).

A relevant role of cAMP-PKA axis in the epigenetic control of gene expression has been proposed. In eukaryotic cells, histone proteins are involved in the control of chromatin structure and remodeling. These are important mechanism(s) that cells adopt to regulate gene transcription (Tessarz and Kouzarides, 2014). Histones undergo to reversible post-translational modifications, such as acetylation. Acetylation/deacetylation cycles of histones are essential processes underlying gene expression and are catalyzed by families of histone acetyltransferases (HATs) and deacetylases (HDACs and sirtuins), respectively (Dekker and Haisma, 2009; Stasevich et al., 2014). Sirtuins have been implicated in a wide range of biological processes, such as transcription, DNA damage repair, and metabolism (Etchegaray et al., 2013). Sirtuin-6 (SIRT6) is a stress-induced gene that belongs to NAD^+^-dependent Class III of histone deacetylases and controls the maintenance of telomere structure and length. By deacetylating histones, SIRT6 regulates genome stability and cell viability (Tennen et al., 2010). Loss of SIRT6 gene induces premature lethality and aging-related degeneration (Mostoslavsky et al., 2006). A link between sirtuins and cAMP signaling has been recently identified. Thus, cAMP stimulates ubiquitylation of SIRT6 protein and its consequent degradation through the proteasome. By reducing the levels of SIRT6, cAMP sustains radiation-induced apoptosis of lung cancer cells (Kim and Juhnn, 2015).

In neurons, cAMP-PKA signaling controls a variety of biological cues, as neurite outgrowth, morphogenesis and synaptic transmission and plasticity (Tasken and Aandahl, 2004). Most of the effects elicited by cAMP are mediated by a transcriptional control of gene expression. A post-translational mechanism of neurite extension which involves the UPS has been recently identified. Thus, neurotrophin-induced activation of PKA promotes praja2-dependent ubiquitylation and degradation of the neurite outgrowth inhibitor NOGO-A. By removing the inhibitory constrain of neurite extension imposed by NOGO-A, PKA-UPS drives a signaling circuit that promotes and sustains neuronal differentiation and synaptic activity (Sepe et al., 2014). In course of neuronal differentiation, phosphorylation of E3 ligases by PKA could also affect substrate recognition, switching the target selectivity between proteins with opposing functions. Thus, neurotrophin-stimulated phosphorylation of Smad ubiquitylation regulator factor 1 (Smurf1), a key component of TGF-β/BMP pathway, reduces degradation of polarity protein Par6 and enhances proteolysis of growth-inhibiting RhoA factor, eventually leading to axon outgrowth (Cheng et al., 2011).Similarly, in cisplatin-treated cancer cells, PKA phosphorylation of Smurf1 prevents degradation of the pro-apoptotic protein Nur77, triggering the mitochondrial apoptotic machinery (Lin et al., 2014).

Dephosphorylation of cellular substrates is mediated by distinct families of protein PPs, (Zhang et al., 2013). Among PPs, protein phosphatase 2 (PP2A) is a conserved Serine/Threonine phosphatase that regulates a wide number of signaling pathways. PP2A is composed of a dimeric core enzyme (structural A and catalytic C subunits), and a regulatory B subunit. In eukaryotes, C and A subunits of PP2A show high degree of sequence similarity, while the regulatory B subunits are highly heterogeneous. The assembly of the three subunits generates different PP2A holoenzymes, whose substrate specificity and intracellular localization are controlled by B subunits (Kiely and Kiely, 2015). PP2A dephosphorylates a variety of substrates, including cAMP-response element-binding protein (CREB). De-phosphorylation of CREB by PP2A attenuates cAMP-induced gene transcription (Wadzinski et al., 1993). PP2A is also involved in a variety of cell functions, as proliferation, differentiation and cell death (Tsuchiya et al., 2014). During apoptosis, PP2A/C subunit is post-translationally regulated by the UPS. Thus, stimulation with tumor-necrosis factor-related apoptosis-inducing ligand (TRAIL) promotes the recruitment of PP2A/C, caspase-8 and Cullin3, a subunit of the cullin family of E3 ligases, into the death-inducing signaling complex (DISC). Within the complex, Cul3 targets PP2A/C for ubiquitylation and degradation by the proteasome. Downregulation of PP2A/C signaling and downstream gene transcription may account for the activation of the apoptotic machinery induced by TRAIL (Xu et al., 2014).

## Dys-regulation of cAMP-UPS in Human Diseases

cAMP signaling is involved in a variety of different biological responses (Formosa and Vassallo, 2014). The complexity of the pathway and the high number of components involved ensure an efficient and a fine regulation of the signal transmission from the site of generation to downstream effectors. Genetic mutations or altered expression of any component of this sophisticated signaling cascade may lead to dys-regulation of the signaling, contributing to the onset and progression of human diseases.

In neurons, cAMP balance is crucial for physiological events underlying learning, memory and loco-motor activity (Gomez et al., 2002; Dell’Acqua et al., 2006). Several studies confirmed the pathogenic role of deranged cAMP signaling in neurodegenerative phenotypes (Satoh et al., 2009; Poppinga et al., 2014). As example, Huntington’s disease (HD) is a genetic neurological disorder characterized by alteration of motor coordination that eventually leads to mental decline and behavioral symptoms. HD is caused by the expansion of a CAG repeat in the Huntington (HTT) gene, which induces accumulation of poly(Q)-expanded mutant HTT protein (mHTT; Labbadia and Morimoto, 2013). Accumulation of mHTT within neurons downregulates cAMP signaling, inhibits CREB-dependent gene transcription and profoundly affects neuronal activity and cell survival (Jeong et al., 2012). Recent evidence pointed to a role of mHTT in UPS-cAMP pathway. In particular, mHTT-mediated proteasome impairment inhibits the proteolytic turnover of R subunits within the striatum, increasing the R/C ratio and favoring reconstitution of inactive PKA holoenzyme. By limiting local activation of PKA, mHTT alters the stability of several proteins and impacts on neurons and loco-motor activity. Under these conditions, forced activation of PKA promotes phosphorylation of components of the proteasome (Rpt6) and rescues the impaired proteasome activity, favoring the removal of mHTT aggregates and improving loco-motor activity (Lin et al., 2013).

The UPS is an important control mechanism of cell growth, survival and metabolism. Removal of tumor suppressors or pro-apoptotic factors could, thus, play an important role in tumor growth. Changes in the levels, subcellular targeting or catalytic activity of the E3 ligases may exert major effect on cell growth and survival. Accordingly, dys-regulation of the UPS has been found in a wide array of human cancer (Landis et al., 1989; Weinstein et al., 1991; Palmer et al., 2000). Recent findings demonstrated that praja2, which regulates R subunit turnover, ubiquitylates and degrades MOB1, a core component of NDR/LATS kinase and positive regulator of the tumor-suppressor Hippo cascade (Hergovich, 2011; Lignitto et al., 2013). Removal of MOB1 by the praja2-UPS pathway attenuates the Hippo cascade and sustains glioblastoma growth *in vivo* (Lignitto et al., 2013). These findings uncover the existence of an intricate interplay between GPCR-cAMP signaling, UPS and tumor suppressor pathways in the control of cell proliferation and tumor growth.

## Concluding Remarks

In the last decades, cumulative evidence uncovered a major role of PKA pathway in the control of important biological activities, ranging from differentiation, growth, metabolism, survival to more sophisticated brain activities. Derangement of the cAMP-PKA pathway has been pathogenically linked to the onset and progression of several neurodegenerative and proliferative disorders. So far, most of the cAMP-PKA effects have been attributed to phosphorylation/dephosphorylation events occurring at distal sites of cAMP generation. Emerging data suggest the existence of a cAMP-driven UPS circuitry that controls the turnover/stability of key elements of metabolic and proliferative pathways. At the same time, mounting evidence indicates that UPS by regulating the stability of components of the cAMP cascade controls directly the strength and duration of cAMP-PKA signals. Dys-regulation of this intricate interface between the cAMP and the UPS may underpin the pathogenesis of human diseases. Therefore, efforts are needed to discover new targets and mechanism(s) connecting the UPS to cAMP-PKA signaling, but also to construct a network that is able to predict and quantify the biological outcome (for example, degenerative versus proliferative phenotypes) of human genetic mutations affecting key elements of these transduction pathways. Understanding the complexity of such regulatory mechanisms and exploring further the biological significance of this kinase-ligase network will help to design novel tools and drugs that selectively restore a perturbed cAMP cascade in various human phenotypes.

### Conflict of Interest Statement

The authors declare that the research was conducted in the absence of any commercial or financial relationships that could be construed as a potential conflict of interest.
